# Geographical patterns and predictors of malaria risk in Zambia: Bayesian geostatistical modelling of the 2006 Zambia national malaria indicator survey (ZMIS)

**DOI:** 10.1186/1475-2875-9-37

**Published:** 2010-02-01

**Authors:** Nadine Riedel, Penelope Vounatsou, John M Miller, Laura Gosoniu, Elizabeth Chizema-Kawesha, Victor Mukonka, Rick W Steketee

**Affiliations:** 1 Department of Public Health and Epidemiology, Swiss Tropical Institute, PO Box, 4002 Basel, Switzerland; 2 Malaria Control and Evaluation Partnership in Africa, PATH, Ferney, France; 3 Departement of Public Health and Research, Ministry of Health, Zambia

## Abstract

**Background:**

The Zambia Malaria Indicator Survey (ZMIS) of 2006 was the first nation-wide malaria survey, which combined parasitological data with other malaria indicators such as net use, indoor residual spraying and household related aspects. The survey was carried out by the Zambian Ministry of Health and partners with the objective of estimating the coverage of interventions and malaria related burden in children less than five years. In this study, the ZMIS data were analysed in order (i) to estimate an empirical high-resolution parasitological risk map in the country and (ii) to assess the relation between malaria interventions and parasitaemia risk after adjusting for environmental and socio-economic confounders.

**Methods:**

The parasitological risk was predicted from Bayesian geostatistical and spatially independent models relating parasitaemia risk and environmental/climatic predictors of malaria. A number of models were fitted to capture the (potential) non-linearity in the malaria-environment relation and to identify the elapsing time between environmental effects and parasitaemia risk. These models included covariates (a) in categorical scales and (b) in penalized and basis splines terms. Different model validation methods were used to identify the best fitting model. Model-based risk predictions at unobserved locations were obtained via Bayesian predictive distributions for the best fitting model.

**Results:**

Model validation indicated that linear environmental predictors were able to fit the data as well as or even better than more complex non-linear terms and that the data do not support spatial dependence. Overall the averaged population-adjusted parasitaemia risk was 20.0% in children less than five years with the highest risk predicted in the northern (38.3%) province. The odds of parasitaemia in children living in a household with at least one bed net decreases by 40% (CI: 12%, 61%) compared to those without bed nets.

**Conclusions:**

The map of parasitaemia risk together with the prediction error and the population at risk give an important overview of the malaria situation in Zambia. These maps can assist to achieve better resource allocation, health management and to target additional interventions to reduce the burden of malaria in Zambia significantly. Repeated surveys will enable the evaluation of the effectiveness of on-going interventions.

## Background

Malaria is an endemic disease in Zambia with a national incidence of 412 per 1,000 inhabitants in 2006. Despite a drop of the reported cases over the last years, it is still the leading cause of morbidity and mortality accounting for 45% of hospitalizations and outpatient department visits with 6,000-8,000 reported deaths [1]. Through the National Malaria Strategic Plan (NMSP) 2005-2010, the Ministry of Health and a network of partners are working toward scaling up effective malaria control interventions with the goal of substantially reducing malaria-related burden, especially among vulnerable populations, such as children under five years of age [2]. Lead by the Ministry of Health, numerous partners, including the Global Fund, the President's Malaria Initiative (PMI), the World Bank and the Malaria Control and Evaluation Partnership in Africa (MACEPA) provide support for scaling up malaria control prevention and treatment services throughout Zambia. The national malaria control programme advocates malaria control through widespread distribution of insecticide-treated mosquito nets, application of insecticides in homes, preventive treatment for pregnant women and effective treatment of infected persons [3]. As part of the programme, 5.3 million insecticide-treated nets were distributed all over the country in the years 2006 and 2007 and 85% households of 15 target district have been sprayed [4]. In addition, in 2007, 60% of all pregnant women got malaria prevention drug and all pregnant woman who visited a public clinic received one insecticide-treated net for herself and every under-five child in the same household [5].

The efforts of malaria reduction require comprehensive baseline maps of malaria risk over the whole country. These maps can guide malaria control at areas of highest need, help limited resources to be distributed more efficiently and assist in the evaluation of the progress of all intervention programmes. Earlier maps of malaria risk in Zambia are based on malaria climatic suitability conditions [6,7], however to date there is no empirical malaria map for the country. Although historical survey data have been compiled by the Mapping Malaria risk in Africa (MARA) project, malaria risk estimates based on these data will not reflect the current situation, which is changing due to ongoing interventions.

In 2006, the Ministry of Health, the Central Statistics Office (CSO), MACEPA, and partners conducted the first national Zambia Malaria Indicator Survey (ZMIS). This is a nationally representative household survey in children under five to assess the coverage of key malaria interventions and to measure malaria-related burden [5]. The survey contains geo-referenced parasitological data for each child that can be used to estimate the malaria risk and draw accurate maps of the current malaria situation in Zambia. In addition, the ZMIS collected information on previous interventions at household level like bed nets or indoor residual spraying (IRS) and socio-economic aspects.

In this paper, the ZMIS data of 2006 were analysed and the first contemporary empirical parasitaemia risk map for the country was produced. The MIS data are expected to be correlated in space due to common environmental exposures, which influence transmission similarly in neighbouring areas. The standard statistical methods assume independence of the observations. To take into account spatial correlation, Bayesian geostatistical models [8] were developed to establish the relation between the parasitaemia data and environmental/climatic predictors of the disease. In addition, the corresponding non-spatial models were fitted for comparison purposes. Environmental data were obtained vie remote sensing (RS). Potential non-linearity in the environment-malaria relation and elapsing time in the effects of environmental predictors on parasite risk were modelled using predictors in categorical scales and fitted by penalized and basis spline (P- and B-splines) functions. Due to large number of model parameters, Bayesian Markov chain Monte Carlo (MCMC) simulation was used for model fit. Model based predictions estimating the risk at unobserved locations were obtained via Bayesian kriging. Parasitaemia risk estimates were linked to population data and the number of children at risk at province level was calculated.

## Methods

### The study area and the ZMIS

Zambia is a republic in Southern Africa. Most parts of the country are high plateau areas covered with savannas and some rivers, valleys and mountains. The country has a tropical climate with the rainy season occurring during December and April.

The ZMIS was carried out from May to June 2006, shortly after the rainy season. The data were obtained from a nationally representative two-stage cluster sample [5]. At the first stage 120 standard enumeration areas (SEA) were randomly selected among about 17,000 SEAs the country is divided. They are located within 58 out of 72 districts from all 9 provinces in Zambia. Within each SEA, a random sample of 25 households was chosen resulting in a total of 3,000 households. A household and a women's questionnaire were conducted with Personal Digital Assistants (PDA). In addition, blood samples in children under five were collected and analysed for anaemia using Hemocue Hb 201 and malaria parasites using Paracheck Pf and thick and thin blood smears. Households were geo-located using the Global Positioning System (GPS). All data were entered in an ACCESS database.

### Socio-economic data

Socio-economic data were obtained from a household survey carried out during the ZMIS. An asset index was created as a weighted sum of 59 different household assets extracted from 17 relevant questions, which were included in the household survey. The weights were calculated by principle component analysis (PCA) on the asset indicators [9]. Then the household asset index was divided into wealth quintiles to create a socio-economic status analysis variable.

### Environmental and population density data

Environmental predictors were extracted from Remote Sensing (RS) sources at spatial and temporal resolutions shown in Table 1. This data are available for free at high spatial and temporal resolution. To take into account the elapsing time between the climatic suitability for malaria transmission and parasitaemia, the climatic data were gathered for different periods (up to one year) prior to the survey starting from May 2005. Day and night land surface temperature (LST), normalized difference vegetation index (NDVI) and land cover types were downloaded from the Moderate Resolution Imaging Spectroradiometer (MODIS) from the U.S. Geological Survey (USGS) Land Processes Distributed Active Archive Center (LP DAAC) [10]. LST data were extracted as averages over 8-day periods at 1 km spatial resolution. NDVI was obtained as a 16-day average at a 0.25 km spatial resolution. Land cover data were available from MODIS for the year 2004 and contained 17 different land cover categories as defined by the International Geosphere-Biosphere Programme (IGBP). They were grouped into five categories, namely wetlands, forests, urban areas, shrublands and others. At each cluster location, the land cover covariate was summarized by the proportion of each land type within a radius of 3 km. During the model fit, the category "others" was dropped from the analysis, to avoid effects of colinearity. Daily rainfall estimates (RFE) were taken from Meteosat 7 satellite images and downloaded from the USGS Famine Early Warning Systems Network (FEWS NET) African Data Dissemination Service (ADDS) [11] at 8 km spatial resolution.

**Table 1:** Source, spatial and temporal resolution of remote sensing (RS) data

Predictor	Spatial Resolution	Temporal Resolution	Source
Day land surface temperature (day LST)	1 × 1 km^2^	8 days	MODIS
Night land surface temperature (night LST)	1 × 1 km^2^	8 days	MODIS
Normalized difference vegetation index (NDVI)	0.25 × 0.25 km^2^	16 days	MODIS
Land cover	1 × 1 km^2^	-	MODIS
Rainfall estimate (RFE)	8 × 8 km^2^	daily	ADDS
Elevation	1 × 1 km^2^	-	USGS
Region (urban/rural)	1 × 1 km^2^	-	HealthMapper
Water bodies (rivers, lakes & wetlands)	1 × 1 km^2^	-	HealthMapper
Population counts	0.5 × 0.5 km^2^	-	Landscan2006

Altitude data were extracted from an interpolated USGS digital elevation model (DEM) [12] available at a spatial resolution of 1 km. The digital maps for three different kinds of water bodies in Zambia (lakes, rivers and wetlands) and urban/rural regions were acquired from the HealthMapper database [13]. The distance from each location to the nearest water body source was calculated in IDRISI 32 (Clark Labs). Estimates of the number of persons living in an area of 500 by 500 square meters were downloaded from the LandScan™ Global Population Database [14] for the year 2006. The percentage of under-five children out of the total Zambia population (17.3%) was obtained from the 2006 data of the U.S. Census Bureau International Database [15].

The coordinates of the SEAs were calculated by the average of latitude and longitude over all household locations within the SEA. These coordinates were used to link the environmental and malaria data. For the purpose of predicting parasitaemia risk at the unobserved locations, a grid with cell size of 3 km by 3 km was overlaid on the Zambia map (resulting in around 100,000 grid cells) and the remote sensing data were also extracted for the centroids of the grid cells.

The MODIS Reprojection Tool (USGS) was used to convert the RS data to geo-referenced maps. Further processing of the environmental data and distance calculation for the water bodies was carried out in IDRISI 32. ArcMap v. 9.1 (ESRI) was used as a mapping tool. Additional data processing was performed in Stata/SE 9.2 (StataCorp LP).

### Statistical models

Most of the climatic RS data are available continuously in time. Depending on the malaria endemicity, the duration of the malaria transmission season and environmental factors, there is an elapsing (lag) time between the climatic suitability for malaria transmission and the occurrence of the disease. To determine this period, which may differ among environmental factors, a lag time analysis was carried out. A lag time is defined as a period prior to the survey during which an average value of the climatic factor was calculated. However, the first 16 days preceding the survey were excluded because parasite development in the mosquito takes around two weeks before the mosquito becomes infectious. For each climatic predictor, a number of analyses variables were created. These variables represent short and long term mean averages of the values of the climatic factor at different lag times. For factors extracted at 1- or 8-day temporal resolution, the lag times were multiples of 8 days (that is 8, 16, ..., 360). For NDVI which was extracted at 16 days temporal resolution, the lag times were multiples of 16 days up to one year. At the end 45 lag time variables were created for rainfall, day and night LST and 22 lag time variables for NDVI.

Bivariate logistic regression models were fitted to assess the relation between the parasitaemia risk outcome and the environmental lag time variables. For each climatic factor the lag time variable, which was further considered in the analysis, was the one giving a model with the smallest Akaike's Information Criterion (AIC). All covariates which were significant in the bivariate analysis at 15% significance level, determined by likelihood ratio tests, were included in a multiple geostatistical logistic regression analysis.

Several geostatistical multiple logistic regression models were fitted to assess and capture potential non-linearity in the malaria-environment relation. These models included covariates (i) in continuous scales (ii) in categorical scales with categories based on quartiles and (iii) fitted by penalized and basis spline (P- and B-splines) curves (see Additional file 1). The model with the best predictive ability was chosen via a model validation procedure. In the geostatistical model specification, spatial correlation was taken into account by including household location-specific random effects and assuming that they derive from a multivariate Gaussian spatial process with zero mean [8]. The covariance between any pair of locations was assumed to be an exponential function of distance between the locations. Covariates and random effects were modelled on the logit scale of the parasitaemia risk parameters. The above geostatistical models have at least as many parameters as the number of locations, but model fit is possible via MCMC simulation methods. Exploratory analyses suggested weak spatial correlation therefore non-spatial models (having smaller numbers of parameters) were also fitted. The model with the best predictive ability was employed to predict the risk at the unsampled locations via Bayesian kriging. Predictions were made over a grid of around 100,000 pixels to obtain a parasitaemia risk map for Zambia.

### Model fit and validation

A random sample of 89 (training) locations was selected for model fit, and the predictive ability of the models was assessed on the remaining 20 (test) locations. The range of distances from the selected test locations to the nearest training location varies from 900 m to 76 km, with a median of about 26 km, suggesting that the locations are representative of the underlying spatial process. Model predictions were compared using the following three approaches [16,17]: i) the model with the highest proportion of test locations falling within the 95% Bayesian confidence (credible) interval (CI) and ii) distance measures between observed and predicted parasitaemia prevalence calculated by the Kullback-Leibler (KL) divergence and an analogue to the χ^2^-test. In particular, for each test location the posterior predictive distribution (PPD) was computed using each fitted model. Based on the PPD, Bayesian confidence intervals were calculated with probability coverage of 95%. Each credible interval was examined weather the test locations were falling within that interval. The model predicting the largest number of test locations within the 95% CI of smallest width was considered as the best one. The KL calculates the mean divergences between the observed and predicted parasitaemia prevalence on the logit scales weighted by the observed value. The analogue to the χ^2^-test is based on the squared distance between the median of the PPD and the observed parasitaemia prevalence divided by the observed prevalence. The model giving the smallest divergence or the smallest distance is considered as the best model.

A mathematical description of the models used is given in Additional file 1. The statistical analysis was carried out in Stata/SE 9.2 (StataCorp LP), Winbugs (Imperial College and Medical Research Council, UK) and in specialized software written by the authors in Fortan 95 (Digital Equipment Corporation) programming language using standard numerical libraries (Numerical Algorithms Group Ltd).

## Results

**Figure 1:**
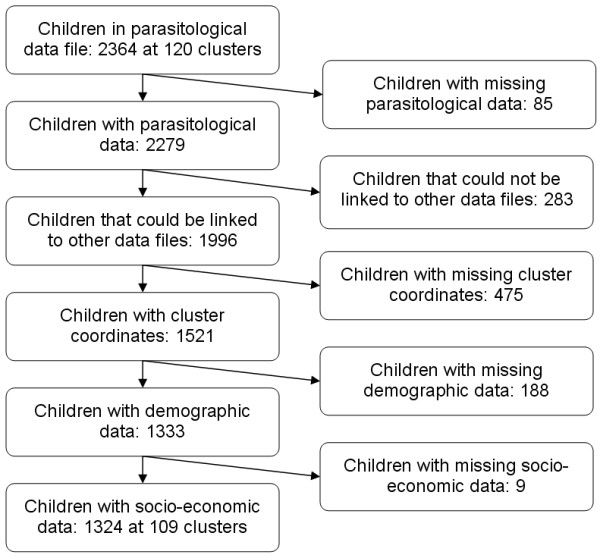
**Study profile of the ZMIS for predicting parasitaemia risk**.

**Figure 2:**
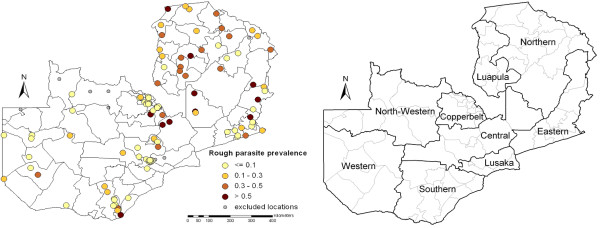
**Observed parasitaemia prevalence (left) and province names (right)**. Observed parasitaemia prevalence within district boundaries at 109 cluster locations used in estimating the distribution of parasitaemia risk in Zambia (left-hand side). The grey dots indicate the 11 clusters that were excluded from the analysis. Province names are given on the right-hand map.

The MIS included 2364 children under five years of age, randomly sampled over 120 cluster locations. However, a sample of only 1324 children at 109 cluster locations had complete parasitological data linked to a geo-located household in order to estimate the distribution of parasitaemia risk. The study profile is given in Figure 1. The sample locations with the observed parasitaemia prevalence are shown Figure 2.

**Figure 3:**
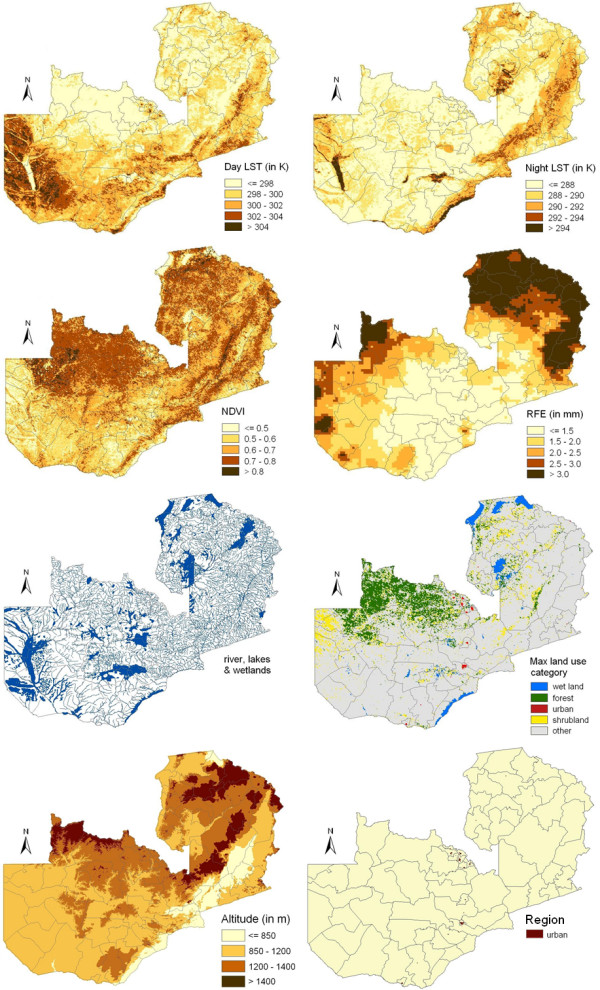
**Spatial distribution of remotely sensed covariates in Zambia**. The climatic factors were summarized over a period preceding the survey indicated by the lag time analysis (day LST, night LST, NDVI, rainfall). The land use map presents the most frequent land use category in a buffer of 3 km around every pixel.

The lag time analysis suggested that the following periods preceding the survey are best (in terms of model fit) for summarizing the climatic factors: 2.7 months for rainfall, 1 month for NDVI, 1.5 months for day and night LST. The geographical distributions of the environmental factors summarized at the above lag times are displayed in Figure 3.

Non-spatial bivariate logistic regression analyses and the likelihood ratio test indicated that all RS factors were significant at 15% significance level (results not presented). All these variables were further included in the geostatistical analysis. Exploratory analysis indicated non-linearity in the relation between the parasitaemia risk and the following environmental predictors: NDVI, rainfall, day and night LST. Various Bayesian multiple logistical regression models (spatial as well as non-spatial) were fitted modelling the non-linearity of the above factors via spline curves or categorical covariates. In addition all models included, land cover types, region type (urban/rural), altitude and distance to the nearest water bodies as categorical covariates. Results on model validation are presented in Table 2. The B-spline models were able to predict correctly most of the test locations (70%) within the 95% CI however the B-spline models produced always 95% CIs with largest absolute widths. In comparison, the spatial P-spline model is able to predict correctly nearly the same proportion of test locations (65%) within a 95% CI with considerably smaller width. Among those models predicting 60% of test locations correctly within a 95% CI, the non-spatial model with the linear terms had the smallest absolute width. This model shows additionally the smallest KL divergence, followed by the spatial P-spline model, and the second smallest χ^2^-value. The spatial model with linear terms has the smallest χ^2^-value. Based on model validation results the non-spatial model with the linear terms was chosen as the final model used for prediction, due to the very good KL divergence and χ^2^-test results and the smallest width of the CIs. This model has also the advantage of a small number of model covariates (compared to the second best model based on spatial P-splines) avoiding over-parameterization problems. The model was employed to predict the parasitaemia risk at unsampled locations and included the following predictors: proportion of each land cover type (excluding the "other" category) within a 3 km radius around the location, categorical covariates for the environmental predictors (altitude, region type and distance to the nearest water bodies) and linear climatic predictors (NDVI, rainfall, day and night LST).

**Table 2:** Model validation summary for the spatial (s) and non-spatial (ns) models

Model	CI (width)	KL	**χ**^2^
Linear (ns)	60% (0.58)	21.49	4867
Linear (s)	50% (0.59)	22.58	4459
Categorical (ns)	60% (0.69)	30.81	6943
Categorical (s)	60% (0.68)	30.33	6143
P-spline (ns)	60% (0.61)	23.11	8194
P-spline (s)	65% (0.61)	22.29	7417
B-spline (ns)	70% (0.72)	27.59	26698
B-spline (s)	70% (0.72)	28.85	26846

Comparison of Bayesian credible intervals (CI) of 95% probability coverage with their corresponding width, Kullback-Leibler divergences (KL) and the χ^2^-test analogue on 20 test locations.

**Figure 4:**
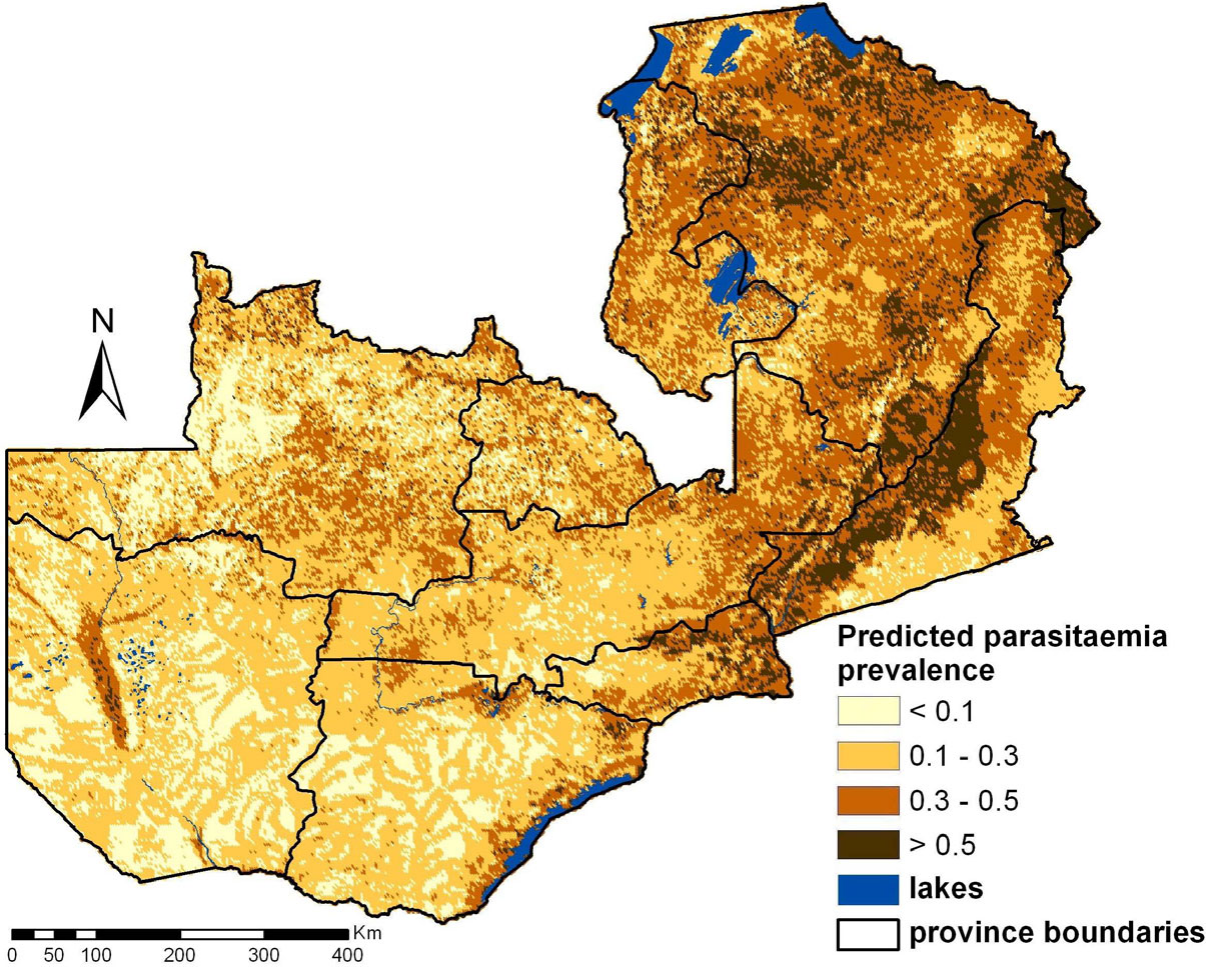
**Predicted parasitaemia risk map for children <5 years in Zambia**. The map is based on a Bayesian logistic regression model with linear terms for day LST, night LST, NDVI and rainfall. The estimates correspond to the median of the posterior predictive distributions computed over 100,000 pixels.

**Figure 5:**
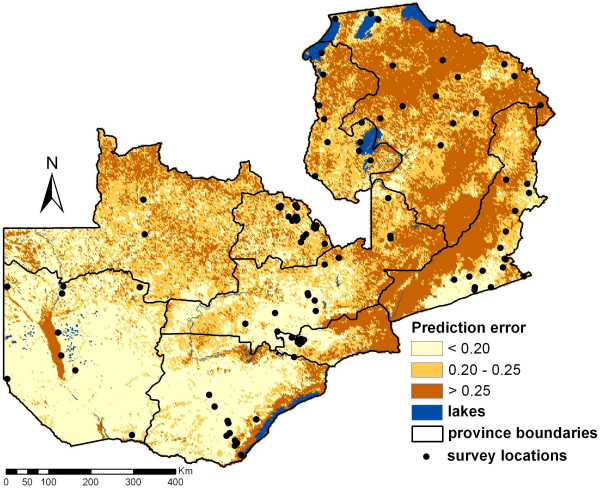
**Prediction error of the parasitaemia risk estimates given in Figure 4**.

Predictions obtained at around 100,000 pixels via Bayesian kriging are shown in Figure 4. The predicted parasitaemia prevalence is ranging between 0.8% and 80.9% based on the posterior predictive distribution, while the observed data vary between 0.2% and 59.8%. The overall prevalence (mean of the prediction for every pixel) is approximately 26.4% with a standard deviation of 15.2% (observed locations: 22.8% with 16.4% standard deviation). Relatively low risks areas (<10%) were frequently predicted for North-Western, Western and Southern province in comparison to the high risk areas (>50%) mainly found in Eastern province and adjacent regions. Estimates of the corresponding prediction error are depicted in Figure 5. The map shows that regions with high prediction errors have high parasitaemia risk. These are mainly areas with sparse survey locations.

**Figure 6:**
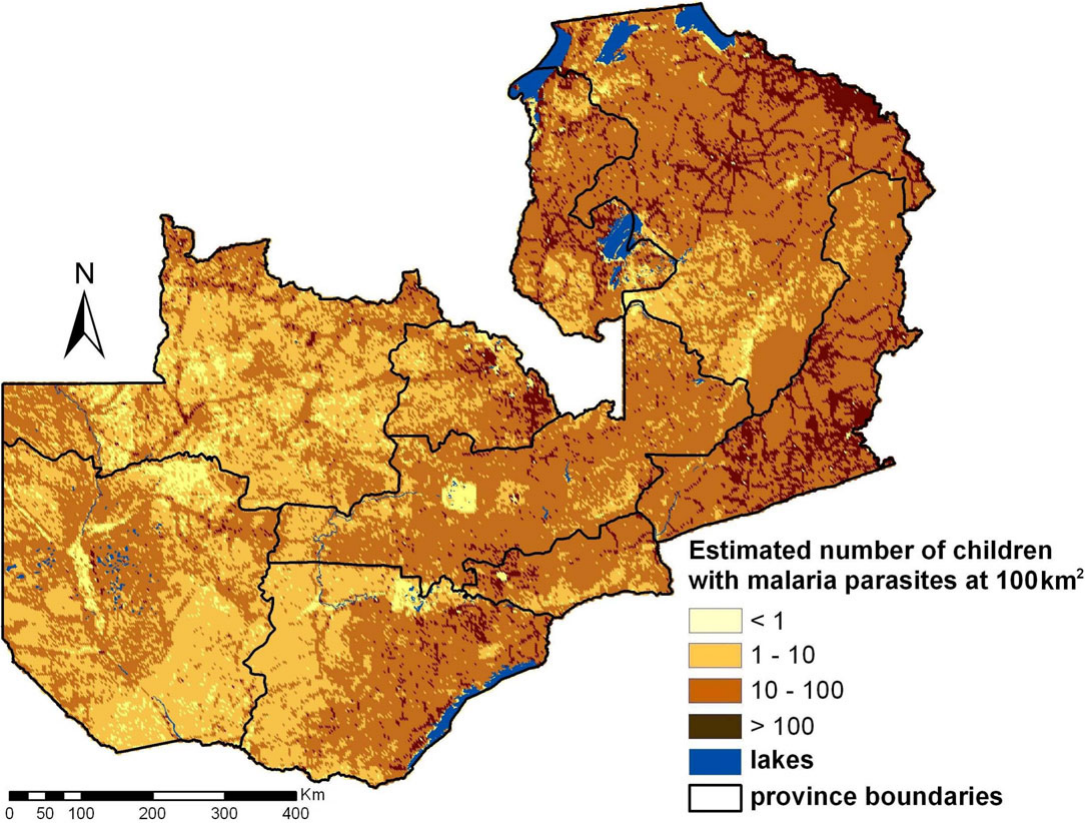
**Estimated number of infected children <5 years per 100 km^2^**.

The parasitaemia risk estimates were combined with the estimated number of children below five years living in the areas of the corresponding pixels to calculate the number of children with parasitaemia. These estimates are presented in Figure 6 in map form and in Table 3 as total counts at province level. Taking into account the population distribution, the average prevalence of parasitaemia risk is approximately 20.0%. Lusaka province has the lowest population-adjusted prevalence level of 7.3% and is the only province with a predicted mean prevalence of <10%. Northern province has the highest population-adjusted prevalence of 38.3% followed by Luapula (30.3%) and Eastern province (27.7%).

**Table 3:** Predicted number of children <5 years with malaria parasites in the blood (per province)

Province	Prev 1 (in %)	Children <5 years	Infected Children	95%CI		Prev 2 (in %)
Central	26.0	182,847	34,572	21,589	50,252	18.9
Copperbelt	23.3	311,317	37,763	18,572	70,719	12.1
Eastern	37.4	240,137	66,614	46,297	87,219	27.7
Luapula	32.0	125,049	37,943	29,039	47,638	30.3
Lusaka	31.8	275,120	20,134	8,121	46,849	7.3
North-Western	21.0	128,935	29,011	16,200	51,616	22.5
Northern	39.1	277,764	106,322	79,379	135,701	38.3
Southern	18.8	243,743	33,430	19,862	53,854	13.7
Western	14.4	147,229	20,321	12,730	30,232	13.8
Total	26.4	1,932,141	386,110	251,789	574,080	20.0

Estimates are based on the mean and the 95% confidence intervals (CI) of the posterior predictive distribution of the non-spatial model with linear terms.

Prev 1: Model based risk estimates

Prev 2: Model-based population-adjusted prevalence

To assess the effects of malaria interventions in Zambia after adjusting for climatic and environmental effects, the above model was fitted again with three additional covariates: socio-economic status of the household, indoor residual spraying within the last 12 months and presence of at least one bed net in the household. These covariates were not used for prediction as accurate estimates of their distribution in Zambia do not exist for 2006. The regression coefficients of this model (multivariate non-spatial) are given in Table 4 together with the bivariate non-spatial logistic regression models. In addition, results of the non-spatial model with linear terms and the second best model (spatial P-spline) are presented. The results of the bivariate regression models reflect significant negative relations with day LST, proportion of urban areas within a 3 km buffer, the region type with urban areas having lower parasitaemia risk, distance to the nearest water body, altitude levels above 1.4 km, socio-economic status (4^th ^and 5^th ^quintile), spraying and presence of bed nets. Positive significant relations were detected with night LST, NDVI, rainfall within the last 2.7 months and proportion of wetlands in the surrounding area. The implementation of the multivariate non-spatial model indicated a loss of significant covariates. The only remaining significant parameter was the presence of at least one bed net in a household which reduces the odds of parasitaemia in children by 40% (CI: 12%, 61%). The final prediction model (non-spatial model with the linear terms) without the additional household specific covariates showed no significant correlation between the predictors and the parasitaemia risk. The non-spatial variance was around 1.7. The P-spline spatial model estimates a ratio of spatial to the total variation of almost 1:2. The minimum distance at which the spatial correlation is lower than 5% estimated by the spatial model is 380 meters (95% confidence interval: 210 m, 3,390 m) which is even lower than the resolution of the grid used for prediction (3 km). This suggests a very weak spatial correlation and supports the choice of the non-spatial model as the one with the best predictive ability.

**Table 4:** Parasitaemia risk predictors of different models

Covariates	Bivariate non-spatial	Multivariate non-spatial	Prediction model	Spatial P-spline model
	**OR (95%CI)**	**OR (95%CI)**	**OR (95%CI)**	**OR (95%CI)**
Day LST	**0.49 (0.43, 0.57)**	0.65 (0.37, 1.15)	0.61 (0.32, 1.17)	*
Night LST	**1.23 (1.09, 1.40)**	1.21 (0.77, 1.88)	1.18 (0.79, 1.77)	*
NDVI	**2.25 (1.90, 2.66)**	1.28 (0.67, 2.73)	1.29 (0.66, 2.77)	*
Rainfall	**1.56 (1.37, 1.76)**	1.21 (0.85, 1.68)	1.18 (0.80, 1.73)	*

Land cover covariates				
Wetland	**1.20 (1.07, 1.34)**	0.97 (0.62, 1.55)	0.98 (0.67, 1.48)	0.72 (0.40, 1.37)
Forest	0.96 (0.84, 1.10)	0.72 (0.43, 1.08)	0.72 (0.43, 1.10)	0.64 (0.38, 0.99)
Urban	**0.35 (0.25, 0.50)**	0.70 (0.38, 1.21)	0.71 (0.37, 1.29)	0.75 (0.33, 1.48)
Shrubland	1.06 (0.94, 1.20)	1.07 (0.76, 1.53)	1.05 (0.71, 1.47)	1.07 (0.72, 1.53)

Region (rural)				
urban	**0.17 (0.11, 0.25)**	0.53 (0.14, 2.03)	0.37 (0.11, 1.13)	0.43 (0.12, 1.50)

Distance to water bodies (<1000 m)				
1000-2499	**0.71 (0.53, 0.96)**	0.73 (0.29, 1.86)	0.71 (0.29, 1.72)	0.55 (0.19, 1.50)
2500-4999	**0.54 (0.38, 0.77)**	0.61 (0.20, 1.55)	0.60 (0.19, 1.72)	0.49 (0.17, 1.42)
≥ 5000	**0.11 (0.04, 0.30)**	0.22 (0.03, 1.40)	0.21 (0.03, 1.39)	0.20 (0.02, 1.93)

Altitude (<850 m)				
850-1199	0.72 (0.49, 1.06)	0.21 (0.03, 1.70)	0.22 (0.03, 1.80)	0.20 (0.02, 1.92)
1200-1399	0.73 (0.49, 1.09)	0.32 (0.03, 3.27)	0.30 (0.03, 3.29)	0.27 (0.02, 4.81)
≥ 1400	**0.48 (0.25, 0.92)**	0.74 (0.04, 10.2)	0.57 (0.03, 7.68)	0.32 (0.02, 7.86)

Socio-economic index (1^st ^quintile)				
2^nd ^quintile	1.06 (0.75, 1.50)	1.21 (0.78, 1.92)	-	-
3^rd ^quintile	0.85 (0.60, 1.22)	1.31 (0.75, 2.23)	-	-
4^th ^quintile	**0.28 (0.17, 0.46)**	0.75 (0.33, 1.75)	-	-
5^th ^quintile	**0.09 (0.04, 0.19)**	0.40 (0.09, 1.56)	-	-

Interventions				
IRS	**0.16 (0.07, 0.36)**	1.73 (0.42, 6.90)	-	-
Bed nets	**0.59 (0.46, 0.77)**	**0.60 (0.39, 0.88)**	**-**	**-**

		**Mean (95%CI)**	**Mean (95%CI)**	**Mean (95%CI)**
Range (in km)	-	-	-	0.38 (0.21, 3.39)
σ^2 ^(spatial error)	-	-	-	0.98 (0.01, 2.77)
τ^2 ^(measurement error)	-	1.77 (0.90, 3.23)	1.71 (0.93, 2.84)	0.82 (0.01, 2.69)

Associations between parasitaemia risk and predictors of the non-spatial model with linear terms and the Bayesian spatial logistic regression P-spline model presented as odds ratios (OR) with their respective 95% confidence intervals (CI).

*: regression coefficients are based on P-spline curves

## Discussion

The ZMIS in 2006 was the first nation-wide malaria survey which combined parasitological data with other malaria indicators such as bed net use, indoor residual spraying and household related aspects. The aim of the survey was to estimate the coverage of interventions and the malaria related burden in children less than five years. However, the MIS data are also a very important source of information for estimating parasitaemia risk at local scales and thus for identifying the high-risk areas that require high intervention coverage and continuous monitoring. Combining parasitaemia risk estimates with population data, the number of infected children can be estimated which can help for better resource allocation, health management and targeted additional interventions to achieve the highest risk reduction for the most populated areas. Repeated surveys will enable the evaluation of the effectiveness of on-going interventions. Already the Zambian Ministry of Health and partners have completed the second ZMIS in the country in 2008 and planning another follow-up survey in 2010.

Prior to the MIS, compiled historical survey data has been used to obtain estimates of parasitaemia risk at high resolution. The mapping malaria risk in Africa (MARA) project was initiated in 1998 with the aim to compile published and unpublished malaria survey data in Africa. MARA is the most comprehensive malariometric database compiling data from 1900 up to date. The MARA data has been analysed using rigorous spatial statistical modelling [17-20] to obtain high-resolution malaria risk estimates at regional and country-level in Africa. However, risk estimates of historical data do not reflect the current malaria situation, which is influenced by on-going interventions. The surveys are not representative as high risk areas tend to be over-represented. In addition historical surveys have been conducted between various locations using different methodologies, including different age groups and carried out at different seasons. On the contrary, MISs do not suffer from these drawbacks. The MIS locations are randomly chosen and the data are available at individual level allowing for estimation of age-specific risk. Another advantage of the MIS data is the household level information available which gives the possibility of differentiating the contribution of climate, socio-economic characteristics and control interventions to the overall parasitaemia risk. If in addition these data are known at high spatial resolution, they can be included in the geostatistical model to obtain more accurate predictions of the malaria risk.

This study created the first contemporary empirical parasitaemia risk map for Zambia. Many existing maps on malaria transmission rely only on rough geographical and climatic iso-lines and expert opinions. Until 1998, none of the maps had a numerical definition, hence the malaria risk maps were not comparable and trustworthy. Then, Hay *et al *[21] produced a climatic map for malaria transmission in Kenya followed by Craig *et al *[22] who developed a climatic suitability malaria risk map for the whole Africa. Up to now there are only a few maps containing also empirical data, the first map of this kind was published in 2000 for Mali by Kleinschmidt *et al *[18]. For Zambia, the first empirical malaria risk map was produced by Hay *et al *[23] and is part of a global risk map based on historical data.

Malaria survey data are expected to be correlated in space. Spatial correlation at short distances is introduced by the transmission process driven by the flight range of the mosquito vector while at wider ranges spatial correlation reflects common exposures to environmental conditions, which influence mosquito survival and longevity. High spatial resolution risk estimation requires prediction at locations where malaria survey data are not available. High-resolution environmental data can be obtained via remote sensing. GIS software has excellent mapping capabilities and it is a very useful tool for processing RS data.

Statistical techniques model the relation between parasitaemia risk and risk factors (environmental, possible interventions, socio-economic factors) via a logistic regression model, which is further used for prediction. Standard statistical methods assume independence of the survey locations. Violating this assumption, when modelling spatially-correlated malaria survey data may lead to imprecise estimates of the risk, the significance of the risk factors and of the prediction error. Similarly, modelling spatial correlation in weakly correlated data increases the number of model parameters and decreases the precision of the estimates. Geostatistical models take into account spatial correlation by introducing an additional parameter (random effect) at each survey location and assume that geographical dependence is a function of distance between locations. Depending on the number of survey locations these models can be highly parameterized and they can only be estimated using Bayesian inference and MCMC simulation. Bayesian geostatistical models have been employed in malaria risk estimation by e.g. Diggle *et al *[19], Gemperli *et al *[20,24] and Gosoniu *et al *[16,17]. However, in this study the Bayesian geostatistical model has estimated very low spatial correlation dropping to less than 5% at distances larger than 380 m, which is lower than the spatial resolution of the pixel size used for prediction (3 km by 3 km). The non-spatial model was superior to the fitted spatial models because spatial correlation is only present at a very local scale indicated by the flight range of the mosquito rather than environmental covariates. Possible reasons might be on-going interventions, which determine mosquito densities and parasitaemia and therefore reduce the influence of environmental predictors on the mosquito.

Previous models of malaria transmission addressed non-linearity between the risk of the malaria-related outcome (in the logit scale) and its predictor solely by categorizing the non-linear covariates. In this study, non-linearity is modelled additionally by using different types of spline curves. The resulting risk estimates suggested that predictions are sensitive to the model fitted. However, model validation indicated that models based on linear terms are superior to non-linear models in Zambia. Even though the P-spline model had the higher predictive ability at 95% CIs, the linear model was considered as the one with the best predictive ability due to the smaller range of these intervals and the results of the KL divergence and χ^2^-measures between observed-predicted prevalence data. This model has also the advantage of an easy interpretability of the regression coefficients for non-statisticians in comparison to the spline curve ones.

None of the regression coefficients of the final prediction model were significant. However, they are needed to determine the mean risk estimate for each location and excluding them reduces heavily the model predictive ability (results have not been shown). The lack of significance of the environmental factors is partially explained by the effects of malaria interventions, which can have stronger influence on the parasitaemia risk than the environmental factors. In fact, the spatial model, which adjusted for different types of interventions, indicated a significant effect of the presence of at least one bed net in a household in reducing parasitaemia risk. Therefore interventions are a major driver of parasitaemia risk in Zambia and including these data in the prediction model would increase the accuracy of model-based risk predictions. Unfortunately, in this study, the parasitaemia risk could not be predicted conditional on bed net coverage because the geographical distribution of bed net coverage was not known for the time of the ZMIS 2006. Intervention data are needed over the entire study area in order to be used for prediction purposes. Since e.g. MACEPA is putting a lot of efforts in scaling up malaria interventions in Zambia, the role of interventions is likely to even increase within the next couple of years. Therefore, compilation of intervention coverage data at high spatial resolution is becoming essential to create reliable risk maps.

The prediction map indicates high variation of parasitaemia risk over the country. In particular, high risk is predicted a stripe from south to east of Zambia which is characterized mainly by low altitude, high NDVI, high day and night LST. The relatively small risk in the south-west of the country might be influenced by low vegetation and rainfall while the small risk in the north-west regions could be due to low day LST and high proportion of forests. It is also possible that malaria interventions have been focused on selected areas like the Zambezi river reducing the parasite prevalence in those areas. It is interesting to note that the maps have been shown to local experts who confirmed the depicted risk patterns. The map of the prediction error of the Bayesian model could assist in improving precision of the parasitaemia risk prediction by identifying the areas of high uncertainty where additional survey locations could be randomly distributed in following MIS's and hence reduce the error and raise the precision of following studies.

The reduced smoothness of the map might be explained by the weak spatial correlation which most likely indicate that interventions counteract the environmental effects. The sampling framework of the MIS may also tend to underestimate spatial correlation because the survey clusters are partially driven by population density, where transmission is largely influenced by interventions. However the drastic changes within small distances are highlighting the importance of the high-resolution maps. If prediction would have been done at smaller resolution some of the high risk areas would disappear and the burden would have been underestimated in these regions. For other purposes, which do not need such high precision, reducing the resolution can be easily done by combining neighbouring pixels.

The average predicted parasitaemia risk over the whole country was 26.4%, however after adjusting for the population size the risk dropped down to 20.0%. It is striking that although the average risk in Lusaka province is the forth highest of all provinces (31.8%), after taking into account the population density the risk reduces to 7.3% indicating that the highest risk areas are the less populated. In fact Lusaka is the province of the lowest population-adjusted risk. The provinces of Copperbelt, Southern and Western have low population-adjusted risks (less than 15%). On the other hand Northern province has the highest population-adjusted parasitaemia risk followed by Eastern province and Luapula. In addition, Northern province has the highest number of infected children less than five years old. Therefore, interventions should be concentrated in the Northern province especially at the north-eastern border and in the middle of the province. Further interventions in Copperbelt and Southern might also have a strong impact in reducing the overall burden since the total number of infected children is as high as in Luapula even though their prevalence is lower than 15%.

## Conclusions

The map of parasitaemia risk together with the prediction error and the population at risk give an important overview of the malaria situation in Zambia. The maps can be used by decision-makers to allocate resources and interventions to reach the most persons in the regions of highest risk to reduce the burden of malaria significantly for Zambia. The ZMIS of 2008 and 2010 will provide very important information on the changes of the parasitaemia risk over space and time and help in the evaluation of the progress of new and established intervention programmes adjusted for environmental drivers of the transmission risk.

## Competing interests

The authors declare that they have no competing interests.

## Authors' contributions

NR analysed the data and drafted the manuscript. PV was responsible for conception and design of the analysis, supervised the implementation and revised the manuscript. JMM participated in designing and coordinating the fieldwork and provided important intellectual content to this study. LG contributed in the analysis and helped to draft the manuscript. ECK gave intellectual content and revised the draft critically. VM enabled the MIS 2006 to occur and has been instrumental in championing the use of this data for guiding decisions. RWS coordinated the fieldwork and reviewed the manuscript. All authors read and approved the final manuscript.

## Supplementary Material

Additional file 1 Additional information regarding model formulation and spline curves. Click here for file
