# Supplementary material for: Geographical patterns and predictors of malaria risk in Zambia: Bayesian geostatistical modelling of the 2006 Zambia national malaria indicator survey (ZMIS)
Source: Malar J. 2010 Feb 1;9:37. doi: 10.1186/1475-2875-9-37 (PMC2845589; doi:10.1186/1475-2875-9-37)
Supplement: Additional file 1 — Additional information regarding model formulation and spline curves. [file 1475-2875-9-37-S1.PDF]

Appendix

Let Y_{ij} be the binary outcome of the parasitological status and p_{ij} the probability of a parasitaemia risk of child j at location i, $i = 1,...,n$. We assume that Y_{ij} arises from a Bernoulli distribution, $Y_{ij} \sim Be(p_{ij})$ and model the associated m covariates $\mathbf{X}_{ij} = (X_{ij1},...,X_{ijm})^T$ on the logit scale, that is $\log it(p_{ij}) = \sum_{l=1}^{m} f_l(X_{ijl};\beta_l)$ where $\boldsymbol{\beta} = (\beta_1,...,\beta_m)^T$ is the vector of regression coefficients and $f_l(\cdot)$ is a function of the covariates which in the case of linearity is defined as $f_l(X_{ijl};\beta_l) = X_{ijl}\beta_l$. We introduce at each location a random effect (error term) ϕ_i and assume that $\Phi = (\phi_1,...,\phi_n)^T$ are realisations of an underlying Gaussian process, $\Phi \sim MVN(0,\Sigma)$ with covariance matrix Σ modelling spatial correlation as an exponential function of distance between locations, that is $\Sigma_{st} = \sigma_\phi^2 R_{st}$ and $R_{st} = \exp(-\rho d_{st})$, where ρ measures the rate of correlation decline. For the exponential correlation function, the minimum distance at which the spatial correlation between locations is less than 5% (range of spatial process) is calculated by $3/\rho$. To measure the remaining non-spatial variation, an additional error term ε_i is introduced into the model, drawn from an independent Gaussian distribution $\varepsilon_i \sim N(0,\sigma_\varepsilon^2)$. The model can thus be written as following:

$$\log it(p_{ij}) = \sum_{l=1}^{m} f_l(X_{ijl};\beta_l) + \phi_i + \varepsilon_i .$$

To account for non-linearity in the relation between the logit of parasitaemia risk and covariates three models were adopted including covariates (i) in categorical scales (ii) as P-spline curves and (iii) as B-spline curves. The spline approaches are able to model a smoothed curve (between the covariate and the parasitaemia outcome) with high order polynomial terms. In particular, P-splines define $f_l(\cdot)$ as $f_l(X_{ijl};\beta_l) = \sum_{k=1}^{K} \beta_l^{(k)} \left| X_{ijl} - s_l^{(k)} \right|^3$ where

$\mathbf{s}_l = (s_l^{(1)}, \ldots, s_l^{(K)})^T$ is a vector of K knots such as $\min(X_l) = s_l^{(1)} < s_l^{(2)} < \ldots < s_l^{(K)} = \max(X_l)$. In this paper the knots were chosen to correspond to the quartiles of the respective covariate. To obtain a smooth curve avoiding overfitting, $f_l(X_{ijl};\beta_l)$ is penalised according to the data [1]. The smoothed cubic B-spline curves define $f_l(X_{ijl};\beta_l) = \sum_{k=1}^{K-3} \beta_l^{(k)} B_{l,3}^{(k)}(X_{ijl})$, where $B_{l,3}^{(k)}(\cdot)$ is a set of basis functions locally defined at $\left[s_l^{(k)}, s_l^{(k+1)}\right)$. $B_{l,3}^{(k)}(\cdot)$ is recursively calculated by:

$$B_{l,t}^{(k)}(X_{ijl}) = \frac{X_{ijl} - s_l^{(k)}}{s_l^{(k+t-1)} - s_l^{(k)}} B_{l,t-1}^{(k)} + \frac{s_l^{(k+t)} - X_{ijl}}{s_l^{(k+t)} - s_l^{(k+1)}} B_{l,t-1}^{(k+1)} \text{ with } t = 2,3$$

where $B_{l,1}^{(k)} = 1$ [2].

To complete Bayesian specification, we assumed for the regression coefficients, independent normal prior distributions with mean 0 and variance 100, while for σ^2 and τ^2 inverse gamma distributions with mean 1 and variance equal to 100. The ρ parameter was considered to arise from a Uniform distribution between 200m and 830km based on the maximum and minimum distances of the ZMIS survey locations. Model fit was carried out via Gibbs sampling, running with two chains. The model was converged before 800,000 iterations. Convergence was assessed by inspection of ergodic averages of selected model parameters. After convergence, the algorithm was run for another 2500 iterations to collect a sample of size 500 (1 sample for every 10 iterations per chain).

1. Crainiceanu CM, Ruppert D, Wand MP: **Bayesian analysis for penalized spline regression using Win BUGS.** *J Stat Softw* 2005, **14**.
2. De Boor: *A Practical Guide to Splines.* Berlin: Springer; 1978.
